# Deep Learning for Flow Sculpting: Insights into Efficient Learning using Scientific Simulation Data

**DOI:** 10.1038/srep46368

**Published:** 2017-04-12

**Authors:** Daniel Stoecklein, Kin Gwn Lore, Michael Davies, Soumik Sarkar, Baskar Ganapathysubramanian

**Affiliations:** 1 Iowa State University, Mechanical Engineering, Ames, 50011, USA

## Abstract

A new technique for shaping microfluid flow, known as flow sculpting, offers an unprecedented level of passive fluid flow control, with potential breakthrough applications in advancing manufacturing, biology, and chemistry research at the microscale. However, efficiently solving the inverse problem of designing a flow sculpting device for a desired fluid flow shape remains a challenge. Current approaches struggle with the many-to-one design space, requiring substantial user interaction and the necessity of building intuition, all of which are time and resource intensive. Deep learning has emerged as an efficient function approximation technique for high-dimensional spaces, and presents a fast solution to the inverse problem, yet the science of its implementation in similarly defined problems remains largely unexplored. We propose that deep learning methods can completely outpace current approaches for scientific inverse problems while delivering comparable designs. To this end, we show how intelligent sampling of the design space inputs can make deep learning methods more competitive in accuracy, while illustrating their generalization capability to out-of-sample predictions.

As the availability and power of modern computing resources has increased, so has interest in the field of inverse problems in science and engineering^1^,^2^. In particular, ill-posed inverse problems for which there is no analytical solution or certainty of a unique solution, but have a tractable forward model, are now more easily solved with modest computing hardware. An example of such a physical system is a recently developed method of fluid flow manipulation called flow sculpting. Flow sculpting uses sequences of bluff-body structures (pillars) in a microchannel to passively sculpt inertially flowing fluid (where 1 < *Re* < 100, for Reynolds number 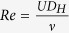, with fluid velocity *U*, viscosity *ν*, and channel hydraulic diameter *D*_*H*_). Specifically, fluid flowing in the inertial regime past a pillar shows broken fore-aft symmetry in the pillar-induced deformation, which laterally displaces the cross-sectional shape of the fluid in some way depending on the channel and pillar geometry, and the fluid flow conditions (described by *Re*)^3^. By arranging pillars in a sequence within a microchannel, the fluid will experience individual deformations from each pillar, resulting in an overall net deformation at the end of the sequence (see Fig. 1 for an illustration of flow sculpting). With sufficient spacing between each pillar in a sequence, the deformation from one pillar will saturate before the flow reaches the following pillar. Therefore, the deformation caused by a single pillar can be viewed as an independent operation on the fluid flow shape, enabling a library of pre-computed deformations to predict the sculpted flow shape for a given pillar sequence. Work by Stoecklein *et al*. demonstrated this forward model with user-guided manual design in a freely available utility “uFlow” (www.biomicrofluidics.com/software.php) to create a wide variety of useful flow shapes^4^.

Flow sculpting via pillar sequences has since been applied to problems in biological and advanced manufacturing fields. For example, polymer precursors can create shaped microfibers and particles^5^,^6^,^7^,^8^, and a pillar sequence can shift fluid streams away from cells in flow^9^. Although novel in their application, these use cases utilize simple micropillar sequence designs (e.g., forming an encapsulating stream, or shifting fluid to one side of a microchannel). More complex fluid flow shapes could lead to powerful new technologies in the aforementioned fields, for example: fabricated microparticles could be designed for optimal packing efficiency, or to focus with directed orientation to specific locations within a microchannel for improved on-chip cytometry^10^; porous hydrogel could be designed to reduce wound healing time^11^, or study cell growth and chemotaxis^12^. These are perhaps more obvious applications of flow sculpting, but as more disciplines and industries are exposed to the technique, new possibilities are expected to abound.

However, microfluidic device design via trial-and-error is tedious, and requires user intuition with the design space. Choosing from many sizes and locations for individual pillars, in addition to their sequential arrangement in a microchannel, produces an enormous combinatorial space of fluid flow transformations. It has also been shown that the space is multi-modal, with many micropillar sequences creating similar fluid flow shapes, making design optimization non-trivial. Clearly, these features (a large *many*-*to*-*one* design space with non-linear fluid transformations) are endemic to many engineering inverse problems, with flow sculpting being a good representative. Thus, manual design of micropillar sequences is generally impractical for most of its intended users, which includes researchers in fields such as advanced manufacturing, biology, bio-sensing, healthcare, pharmaceuticals, and chemistry^13^,^14^,^15^. This drives the need for an automated solution to the inverse problem: designing a micropillar sequence that produces a desired fluid flow shape. To date, there are two automated approaches in literature: heuristic optimization via the Genetic Algorithm (GA)^16^,^17^ and deep learning via trained Convolutional Neural Networks (CNN)^18^. While the GA capably optimized existing microfluidic devices and explored novel flow shapes, there exist a few drawbacks to its use. GAs require well-crafted cost functions specific to different problems, necessitating that the user have knowledge of programming and optimization. The GA is also a stochastic method, with no guarantee of finding global optima using a finite number of searches. For flow sculpting, this leads to excessive runtime (as much as 2 h), which makes swift design iterations difficult^17^. On the other hand, the application of deep learning shown by Lore *et al*. operated with an extremely quick time-to-result (≈1 s), but lacked in accuracy comparable to the GA^18^. We seek to carefully improve and demonstrate the capabilities of deep learning in a comprehensive manner for flow sculpting with this work. Our study also reveals fundamental insights into training strategies for machine learning of engineered systems, which aids in providing best practices for analogous engineering inverse problems.

A key motivation in the use of deep learning for design in flow sculpting is the ability to generalize patterns and associations between input and output data into a globally effective mapping^19^. That is, a properly trained deep neural network (i.e., with appropriate model structure and regularization, sufficient training data, and carefully chosen hyperparameters) does not strictly memorize the relationship between local regions in the input space to their corresponding regions in the output space, but creates a function that maps input data to the output space based on the learned hierarchical features and rules of the underlying physical system (i.e., rote memorization vs. actual learning and generalization). This allows a trained neural network to operate in as-yet unseen regions of the input space. This is especially of interest for flow sculpting, as a completely exhaustive search for tailored fluid flow shapes is computationally infeasible. This is owed to the combinatorial complexity in the design of a pillar sequence. Previous work has performed effective design in choosing among 32 possible pillar configurations (four pillar diameters and eight lateral locations in the channel), using 1–10 total pillars in a sequence^4^. Thus, a fluid flow shape created from a 10-pillar sequence exists among 32^10^ ≈ 10^15^ possible combinations, making this a difficult combinatorial problem. On the other hand, a trained neural network could map a large portion of the design space from a significantly smaller sample. The rapid feedback in using a deep learning tool - generally a matter of seconds - also makes for a user-friendly experience. Moreover, the design space will continue to grow as additional microfluidic components are added to the flow sculpting toolset, for example: half-pillars, steps, and curved channels can also be used to deterministically sculpt fluid flow. Thus, it is important to have a design utility that maintains parity with new flow sculpting tools without requiring extensive re-training and fine tuning, which aligns with the strengths of deep learning.

We speculate that an impediment to performance in deep learning techniques for design in flow sculpting is the *many*-*to*-*one* design space. That is, there may be many solutions (pillar sequences) that produce a desired fluid flow shape. Consider the set all possible pillar sequences ***s***_***i***_ as the space 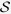, and their corresponding fluid flow shapes ***o***_***i***_ as the space 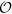, with a forward model *f* that maps a specific realization 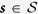 to 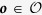, i.e. 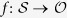. A deep neural network attempts to construct an approximation to *g* = *f*^−1^, with the inverse function *g* mapping 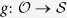. During training, a deep neural network that has been shown a pillar sequence and fluid flow shape pair (***s***_**1**_, ***o***_**1**_) may be trained on another flow shape, ***o***_**2**_, that is extremely similar to ***o***_**1**_ and thus occupying the same space in 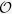. However, the pillar sequence ***s***_**2**_ that produces ***o***_**2**_ could be entirely different from ***s***_**1**_, and therefore very far apart in 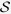. Hence, the *many*-*to*-*one* mapping 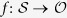 could make effective training quite difficult.

Appropriate selection of training data and an understanding of what constitutes “good” training remain open challenges in modern applications of machine learning^20^. However, unlike traditional problems in machine learning - image classification or speech and handwriting translation, for example, where training data attempt to sample an unbounded and highly variable space - the domain of flow sculpting is finite (though extremely large). Furthermore, the space of sculpted flows presents a natural metric (i.e., binary images with sculpted flow and co-flow) that enables efficient characterization of the data space. This offers a unique opportunity to explore how domain knowledge and the choice of sampling can influence high-level decision making in a deep learning model. While our focus is clearly on the flow sculpting problem, the issues raised here would tend to appear in other inverse problems. Similar critical scientific optimization problems, such as robotic path planning, material processing, or design for manufacturing can benefit from the insight on intelligent sampling gained here. We explore a sampling method for choosing training data known as High Dimensional Model Representation (HDMR)^21^, and analyze the space 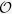 using dimension reduction via Principal Component Analysis (PCA). Our analysis includes a parameter study on training set size, along with several out-of-sample studies to demonstrate deep learning’s capability to generalize for this complex *many*-*to*-*one* problem. We also test the hypothesis that a training set with a more uniform distribution in 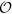 will lead to a more accurate model.

## Results and Discussion

### Flow Sculpting Physics

**Figure 1:**
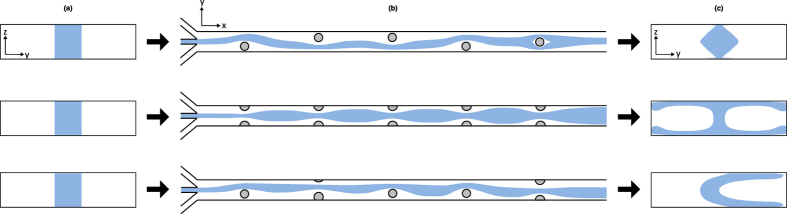
Illustration of three different flow sculpting devices that modify the same inlet fluid flow. (**a**) For a given inlet flow configuration (shown here in a cross-sectional view with the middle-fifth of the channel containing the sculpted fluid, colored blue), arbitrary sequences of pillars (**b**) (shown as top-down views of three different microchannels) can purposely sculpt the cross-sectional shape of fluid, yielding a net deformation (**c**) at the outlet of the channel.

The concept and implementation of inertial fluid flow sculpting via pillar sequences has been previously investigated by the work of Amini *et al*.^3^,^13^ and Stoecklein *et al*.^4^,^16^,^17^. We will briefly elaborate on the generalities of flow sculpting to clarify the application for this work. Most microfluidic devices typically employ low-*Re* fluid flow, known as Stokes flow, such that *Re* ≈ 0^22^ (in this case, the characteristic length that defines *Re* is the microchannel hydraulic diameter). This flow regime is generally achieved via small length scales and low flow rates, and is highly laminar, easily controlled, and well predicted. One consequence of Stokes flow is that fluid flowing past an obstacle exhibits no inertial effects. Therefore, in Stokes flow past an obstacle in a microchannel, fluid will return to its original location in the channel cross-section, with no apparent displacement. However, by increasing *Re*, inertia in the fluid begins to break this symmetry around the ostacle, inducing a deformation in the fluid as it flows past the pillar. This effect was first shown in a confined microchannel by Amini *et al*., who determined that, in using a pillar as an obstacle, post-pillar time dependent effects do not appear until *Re* > 100^3^. Hence, flow conditions of 1 < *Re* < 100 define the operating regime for which fluid flow past a bluff-body obstacle will exhibit laminar behavior, but is predictably deformed in a time-independent manner. The nature of the deformation will depend on pillar diameter, location in the channel, flow physics (*Re*), and channel geometry (aspect ratio, for a rectangular channel). The idea of flow sculpting is to leverage single-pillar flow deformations as independent operations on the cross-sectional fluid flow structure, and piece multiple pillars together to create a more complex net deformation at the end of the pillar sequence. An illustration of inertial flow sculpting is shown in Fig. 1, where three different sequences of five pillars each deform the same inlet flow pattern into distinct flow shapes at their respective outlets.

A necessary condition for flow sculpting is that pillars must be placed far enough apart such that each individual pillar’s flow deformation saturates before the fluid arrives at the subsequent pillar, preventing cross-talk between two pillars. The prevention of cross-talk enables a powerful computational shortcut for simulating flow sculpting devices, as complex flow fields for single-pillar configurations - which require significant computational effort to acquire - need only be solved once before their subsequent use in simulating a pillar sequence. That is, rather than solving the Navier-Stokes equations for fluid flow within an entire multi-pillar device, pre-computed solutions for the individual pillars that comprise the device can be assembled in real-time for an accurate prediction of sculpted flow. Provided that the microchannel dimensions and Reynolds number for each pre-computed flow deformation - stored as a 2-D “advection map” - match, an arbitrary arrangement of sufficiently spaced pillars can be well predicted by the concatenation of their respective maps. This informs the creation of a pre-computed library of advection maps for a variety of pillar configurations, which can be easily utilized with modest computing power for flow sculpting design. Additional details for the implementation of the forward model is in the Methods section.

### Deep Learning Framework

Deep learning, a branch of machine learning, emphasizes the use of multiple levels of abstraction of data. It achieves this by using hyper-parametrized model structures composed of nonlinear transformations of data^23^,^24^. Deep learning methods have been a very attractive option to researchers for data dimensionality reduction^25^, collaborative filtering^26^, feature learning^27^, topic modeling^28^, and solving classification problems^29^. Recently, deep learning has also gained immense traction in the science and engineering community as a tool for data-driven decision making systems. Examples of its wide applicability across scientific disciplines include steady flow approximation^30^ and early detection of combustion instability^31^ for engineering; low-light image enhancement for autonomous mobile systems^32^ and policy learning and reward estimation^33^ for decision and support; high-throughput plant phenotyping^34^ and modeling specificity of DNA-protein binding^35^ for biology and plant sciences; and in high-energy physics, researchers have used deep learning to search for exotic particles^36^ and modes for Higgs Boson decay^37^.

**Figure 2:**
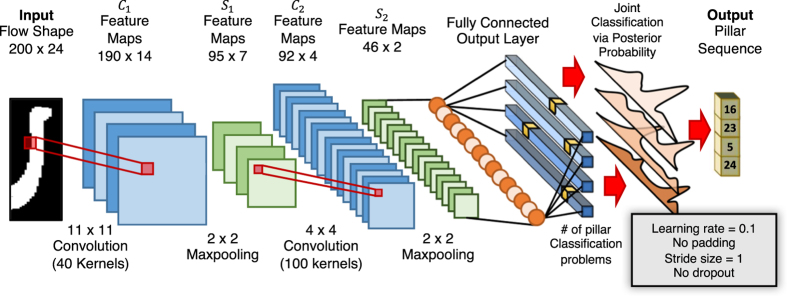
Schematic of the Convolutional Neural Network (CNN) used in this work. The input image on the left consists only of pixels from the top-half of a microchannel simulation, due to the top-bottom symmetry implied by the pillars spanning the full channel height. Note the posterior distributions in the joint classification layer which determine the predicted pillar sequence via maximum a posteriori probability estimate.

We make use of a Convolutional Neural Network^38^ framework and pose the inverse problem in flow sculpting as a simultaneous multi-class classification problem. A schematic of this framework is shown in Fig. 2, with the images as inputs and their corresponding pillar sequences matching to a fixed number of classifier nodes at the output. Note that for the deep learning framework, which is intended to solve the inverse problem, by building an approximation of the map 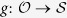 it is implied that the fluid flow shapes in 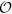 become inputs for the CNN, while pillar sequences in 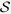 are the output. Convolutional neural networks use feature maps to preserve spatially local correlations within an image, therefore exploiting the 2D structure of input data to improve detection, classification, and prediction over typical neural networks. These feature maps are obtained by convolving filters, which comprise an early hidden layer in the neural network, over the entire image. The feature maps then undergo a process called maxpooling, which is a method of downsampling by overlapping sequential filters such that important information is retained from the feature maps while reducing the dimensionality of the system for subsequent layers. Our CNN repeats this process over smaller filters before feeding the reduced mapping into a fully connected output layer, which operates as a classifier for a pillar sequence corresponding to the flow shape. Every pillar in the output sequence is classified using integer values 1–32, choosing from 32 possible types of pillars. Each type of pillar is defined by a specific size and location: four diameters (*D*/*w* = {0.375, 0.5, 0.625, 0.75} for pillar diameter *D* and channel width *w*), and eight different locations in the channel (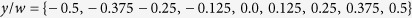, where *y*/*w* = 0.0 is the center of the channel). This library is created by the same experimentally validated simulation technique used by Stoecklein *et al*.^16^,^17^ and Lore *et al*.^18^, which is explained in more detail within the methods section of this work. We use this CNN in several experiments outlined below.

### Principal Component Analysis

We utilize Principal Component Analysis (PCA) to characterize the space 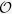. PCA is a method of linear dimensionality reduction that creates an orthogonal basis within a dataset’s high-dimensional space, which optimally explains the variance of every observation in the dataset^39^. Once this basis is constructed, every observation from the original dataset can be shown to be a linear combination of each high-dimensional axis (“principal component”) of the new basis. Importantly, the principal components are ordered in terms of variance explained within the original dataset, i.e., the 1^st^ principal component explains the most variance within a dataset, the 2nd principal component explains the second-most variance, and so on. PCA aids in high dimensional data visualization, as the degree to which an observation uses each principal component provides a mapping onto the newly constructed high-dimensional basis. This means that the original high-dimensional dataset can be projected onto an *n*-D orthogonal space (“PCA-space”), where the coordinates of a projected observation from the dataset (flow shape images, for this work) shows how that data aligns with an axes’ principal component. Therefore, flow shape images that project to nearby regions in the PCA-space are similarly explained by the principal components of those axes, implying that they are similar flow shapes. Conversely, points far apart in the PCA-space imply that the compared fluid flow shapes are more dissimilar.

**Figure 3:**
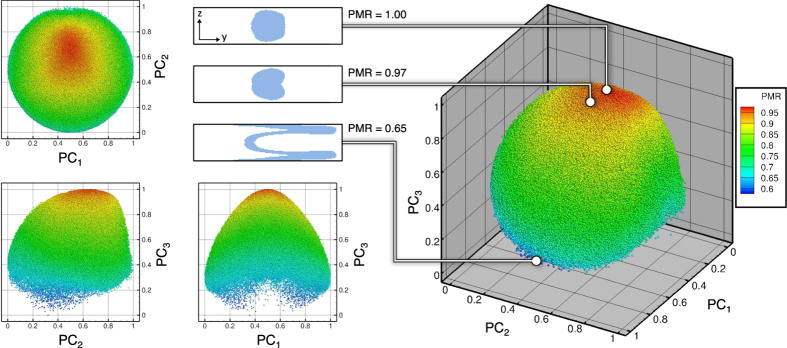
Visualization of the space 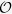 via PCA. This illustration shows the projection of a training set of 150,000 images (chosen by random sampling of 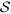) onto its PCA-space, with the first three principal components as the axes *PC*_1_, *PC*_2_, and *PC*_3_, and every point corresponds to an image in the dataset. Each point (image) is colored with a measure of similarity, the Pixel Match Rate (PMR), to a single image in the set (arbitrarily chosen as the image with the largest *PC*_3_ coordinate). A high PMR (red) means the flow shape is more similar to the selected image, while a low PMR (blue) is less similar. Inserted are the selected target flow shape image (top) and two other sample images, each with their respective PMR value to the target image and indicated location in the PCA-space.

An example of PCA projection of a training set of flow sculpting image is shown in Fig. 3. The statement above concerning proximity in the PCA-space is demonstrated, where a target image is selected near the top of the 3-D grouping. All other fluid flow shapes are compared to this target image using the Pixel Match Rate (PMR), a measure of image similarity discussed below which compares the number of matching pixels of two images (a higher PMR means two images are more similar). For this projection, approximately 44% of the variance in the high dimensional data is explained by the first three principal components. While this does not explain all variation in the data, it is clear from Fig. 3 that flow shapes which project to similar locations in the PCA-space are also similar in their full dimensionality, and vice versa. We use the PCA-space as a visually interpretable representation of 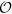, and utilize this to reason about the performance of our deep learning tool, and perform 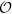-sampling, discussed below.

### High-Dimensional Sampling

Intuitively, an effective tool for design in fluid flow sculpting should have good coverage of the space 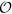. However, knowledge of this space is difficult to come by, as the combinatorial possibilities are immense (for a pillar sequence size *n*_*s*_, the number of possible combinations 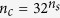). Additionally, the inlet fluid flow design - which dictates fluid flow shape as much as a pillar sequence - is entirely arbitrary, depending on the number of channels joining at the microchannel inlet and their respective flow rates. The current implementation of deep learning for the flow sculpting problem holds the inlet design constant, and trains for pillar sequences of fixed length. Thus, while an ideal training set has completely uniform coverage of 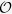, and despite the speed of the forward model, generating and making practical use of such a set for each length of pillar sequence and inlet configuration is computationally infeasible. And, as previously mentioned, the toolset in flow sculpting will likely grow to include curved channels, symmetry-breaking half-pillars and steps, and multi-fluid optimization^40^. Therefore, intelligent sampling on the space 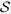 is still a priority. Previous methods of sampling attempted uniform coverage of 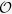 via uniform sampling of 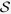, through uniform-random and quasi-random Sobol sampling of *n*_*s*_-dimensional spaces in 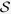 for pillar sequences of length *n*_*s*_^18^.

Here, we apply a technique for multivariate representation known as High-Dimensional Model Representation (HDMR). In HDMR, a high-dimensional input variable 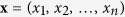 with a model output *h*(**x**) is expressed as a finite hierarchical expansion:


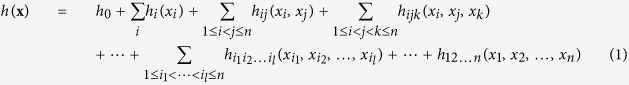


where *l*^*th*^ order component functions 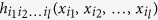 give outputs of *f* evaluated for contributions by *l*-order sampling of **x**. That is, *h*_0_ is a scalar value as the mean response to *h*(**x**), *h*_*i*_(*x*_*i*_) represents each variable *x*_*i*_ independently (holding all other variables constant), and so on. 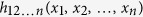 contains residual contributions for permutations of all variables *x*_*n*_. In many cases, a high dimensional system can be well described using up to the 2nd order terms^21^:


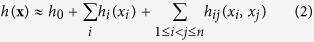


We use this expression to hierarchically sample 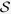, choosing *k* points along each chosen dimension of **x** such that we limit the training set size to be comparable to the pseudo-random and quasi-random sampling used by Lore *et al*.^18^, which comprises of 150,000 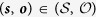. The pseudo-random, uniformly distributed sampling is accomplished via *mt19937ar* Mersenne twister in MATLAB^41^, while the quasi-random sampling was accomplished via Sobol sequences^42^, which seek to maximally separate the sampled points in a deterministic manner in order to prevent clustering or gaps. We neglect the mean response term in the HDMR expansion, as there is no effective “mean” pillar. Since the 1st and 2nd order terms sample a limited number of points in each dimension (choosing one of 32 pillar configurations at each point), there is a choice as to what pillars will be chosen as constant for the rest of the sequence. We desire statistical metrics on the use of HDMR for a fixed *k*, so we randomly pick *k* points in the *i*^*th*^ dimension.

**Figure 4:**
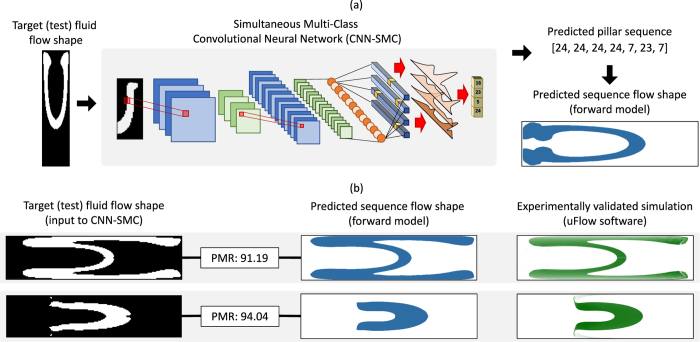
(**a**) The workflow for testing the trained deep learning tool: target flow shapes from testing datasets are used as inputs to the Convolutional Neural Network classifier, which creates a predicted pillar sequence as its output. The predicted pillar sequence is then used with the forward model to create a predicted sequence flow shape. This flow shape is then compared to the original target fluid flow shape using the Pixel Match Rate (PMR). (**b**) Two examples of target flow shapes and their predicted sequence flow shape, with PMR values, along with simulations of the predicted pillar sequence using the experimentally validated software uFlow (www.biomicrofluidics.com/software.php).

For each pillar sequence that is predicted during testing, we use the forward model to create a corresponding flow shape 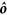 and then compare that to the target image ***o***_***t***_ from the testing set (see Fig. 4). Thus, while the classification error during training is based on label error (that is, correct prediction of the pillar sequence), we judge model performance on whether the trained neural network effectively solves the inverse problem. The metric for performance is a per-pixel norm, the Pixel Match Rate (PMR), which is defined for a target image ***o***_***t***_ and a predicted image 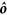 as:


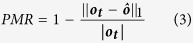


The PMR can vary from 0 (no pixels in common between prediction and target) and 1.0 (a perfect match), but a threshold for a successful result is up to the microfluidic practitioner. For example, if the goal is to simply displace bulk fluid without care for the overall shape, a PMR greater than 0.8 may be suitable. However, if the user requires fine details in the prediction to match the target, a PMR of 0.9 and above is an appropriate threshold. More generally, a PMR of 0.85 is a good result. Our goals are to determine the effectiveness of HDMR in sampling flow sculpting’s input space 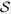, and we use PCA to both characterize these results and test sampling of the flow shape space 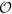 as a method of improving performance. To accomplish this, we conducted three main experiments: (1) a study on training set size, (2) out-of-sample testing using 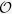-sampling, which selects training and testing data using PCA, and (3) uniform output sampling, which uses PCA for the creation of training data with a uniform distribution on 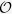. The dimension of the pillar sequence space 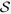 corresponds to the number of pillars used to sculpt flow in a microfluidic device, and in practice would depend on fabrication/design constraints. Typical designs have used between 1–10 pillars, in an attempt to mitigate issues with transverse mass diffusion and increased pressure requirements^16^. For this work we elected to keep a constant pillar sequence size of *n*_*s*_ = 7, as this provides a combinatorially complex space (≈34 billion pillar sequences in choosing from 32 different pillars), while remaining computationally tractable. For the flow shape space, 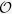, we match the dimensionality of 200 × 24 images used by Lore *et al*.^18^.

#### Training set size study

**Figure 5:**
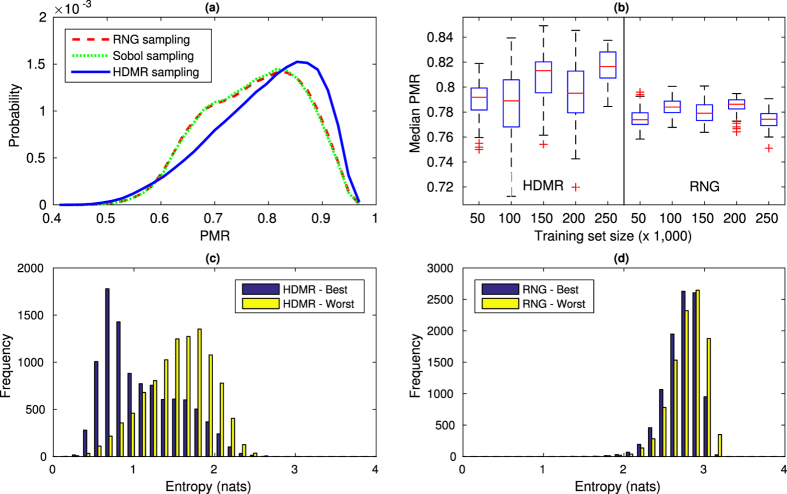
(**a**) Performance of uniform-random (RNG), quasi-random (Sobol), and HDMR sampling on 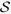 for the creation of training sets of 150,000 flow shapes, with a uniform-random sampled test set of 10,000 flowshapes. The classification was for 7-pillar sequences. (**b**) Performance of HDMR sampling and pseudo-random (RNG) sampling vs. training set size, from 50,000 to 250,000 images in increments of 50,000, with the same testing set as in (**a**). Error bars indicate standard deviation calculated from 50 randomly generated training sets. Note that while RNG sampling can easily generate additional “random” sets of training data, randomly selecting values for *k* sampling indices in HDMR could result in clustering that greatly impairs performance. The distribution of posterior entropy for the CNN models in testing performance of the best and worst (**c**) HDMR and (**d**) RNG training sets are shown for training set size of 150,000. HDMR sampling shows far lower entropy, even with decreased performance, indicating strong model confidence and significant disturbance rejection capability.

The purpose of this study is to directly compare HDMR to previously used methods for sampling 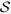, and observe accuracy-scaling trends with training set size. The testing set used was of 10,000 flow shapes randomly sampled from 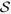. Performance of uniform-random (RNG), quasi-random Sobol, and HDMR sampling with a training set size of 150,000 is shown in Fig. 5(a). As the quasi-random Sobol sampling does not perform significantly differently from uniform random sampling, we will drop Sobol sampling from future analyses, and compare only uniform random sampling with HDMR sampling. A clear shift in the median PMR of roughly 2.5% from 0.79 (RNG) to 0.81 (HDMR) is observed, along with a more left-skewed distribution in favor of high PMR values. In Fig. 5(b), the training size study shows that although the randomly generated HDMR sets have greater variance in data, there is a perceivable increase in performance with larger training set size. RNG sampling shows tighter variance, but no discernible improvement with larger training sets.

We also analyze performance by computing the Shannon entropy of the posterior distribution at the decision (top) layer of a CNN model (as shown in Fig. 2) for a test example during the inference process. Entropy was computed for the best and worst performing training sets for HDMR and RNG sampling with a training size of 150,000 images to show contrast between the sampling methods and their performance. Results are shown in Fig. 5(c,d), where models trained with HDMR sampling exhibit significantly reduced entropy in their predictions, even in the case of the worst-performing HDMR model (mean entropy of 1.09 and 1.55 nats for the best and worst HDMR sampling, compared to mean values of 2.73 and 2.79 nats for the best and worst RNG sampling). A low entropy posterior suggests that the model has a higher confidence in decision-making (in this case, classification of appropriate pillar configuration given the desired flow shape). This also signifies that the corresponding training data set is representative enough with respect to the test sample. At the same time, a model that is able to consistently produce low entropy posteriors generally has a better noise and disturbance rejection capability. This can be argued from the fact that there is a low chance of switching classification decision (when it is made based on the maximum a posteriori, MAP principle) under small disturbances either numerically or in the input. Hence, such a machine learning model is typically preferred in most applications.

#### Out-of-sample study

**Figure 6:**
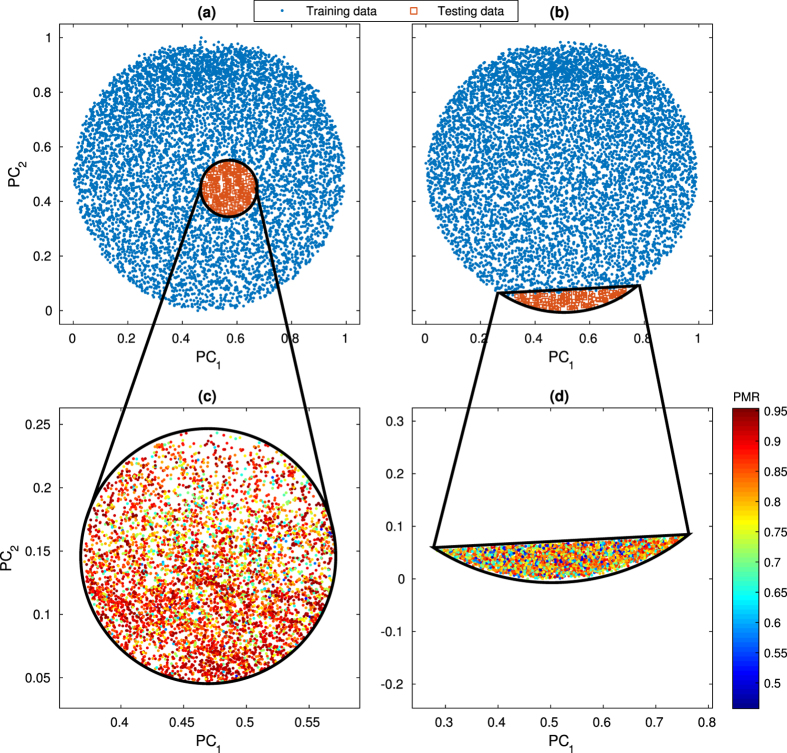
Illustration of out-of-sample testing using (**a**) *interior*-*hole* 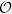-sampling and (**b**) *exterior*-*chord* 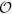-sampling. In each case, data is extracted from a training set based on its projection onto the 2-D PCA-space, leaving a substantial gap in image space coverage where the trained model is then tested. Despite this deficiency, performance is not necessarily hindered, as there are still successful predictions from the trained model as shown in the example (**c**) *interior*-*hole* and (**d**) *exterior*-*chord* test sets. Points in (**c**,**d**) with a higher PMR value (and therefore better prediction) are red, while a lower PMR value is blue.

**Figure 7:**
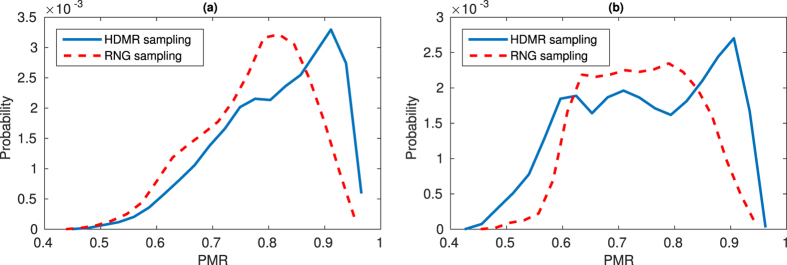
Distributions of out-of-sample results using (**a**) interior-hole and (**b**) exterior-chord sampling for test data extraction from the training set. Note that although performance is generally shifted from the in-sample testing of the training set size study, there are still successful predictions above 85%, especially for HDMR.

To study deep learning’s ability to make predictions beyond the training data, we used a 2-D PCA projection to sample from given training data through either *interior*-*hole* 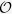-sampling (implying interpolation within the training data) or *exterior*-*chord* 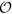-sampling (implying extrapolation outside of the training data), and used these data as test sets (see Fig. 6(a,b)). Since the fluid flow shapes in a given region of PCA-space are similar, eliminating these images from the training set leaves a complementary set of considerably different flow shapes for the neural network to learn from. Thus, for the trained model to succeed, it must truly “learn” the underlying features of the physical system (as opposed to “memorization” of the input/output mapping). For each out-of-sample type (*interior*-*hole* and *exterior*-*chord*), we sampled 50 random locations and created complementary sets of training/testing data for both HDMR and RNG sampled training data. In both scenarios, the trained models clearly have some successful predictions of pillar sequences for target flow shapes outside of the given training data (see Fig. 6(c,d)). This is a clear demonstration of deep learning’s ability to integrate learned features with a mapping that is useful for out-of-sample predictions. Combined distributions of performance are shown in Fig. 7, which relay a general reduction in performance compared to in-sample testing from the prior training set size study (Fig. 5). However, for both types of out-of-sample testing, use of HDMR for training data generation shows a clear increase in successful predictions for which PMR > 0.85. These results imply that while effective coverage of the design space is important for deep neural network training data, the manner by which the data is sampled also plays a crucial role.

#### Uniform output sampling study

**Figure 8:**
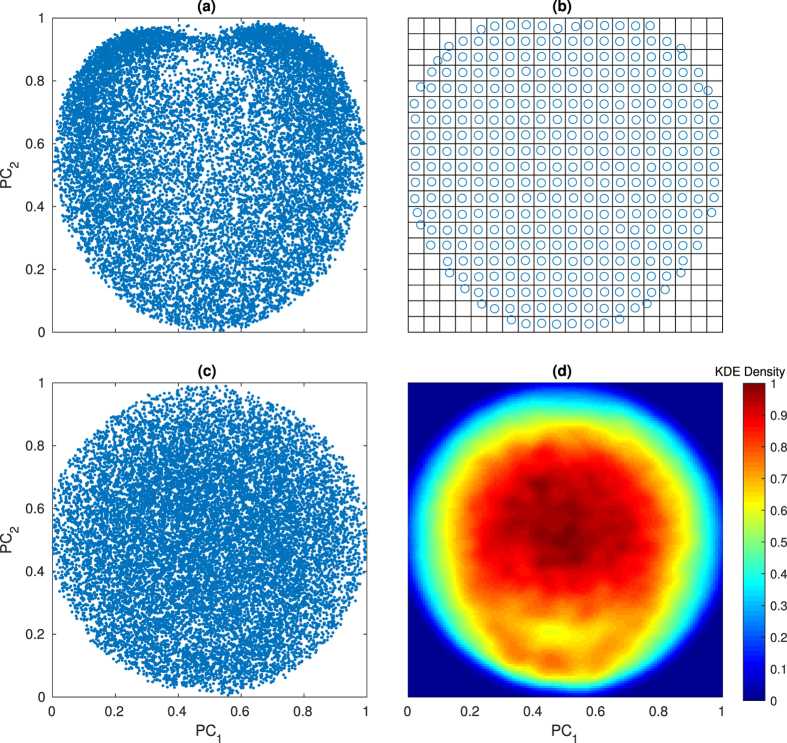
Demonstration of uniform *output* sampling. (**a**) An arbitrary distribution of points in n-D PCA-space can be uniformly sub-sampled (**b**) by isolating sampled points within individual n-cubes, the size of which depends on the level of discretization. By composing 50 sets of 150,000 images randomly sampled from 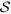 (for a total of 7.5 million images), *output* sampling in 4-D PCA-space is used to create a more uniform set of 150,000 images (**c**), as seen in (**d**) with an applied bivariate Kernel Density Estimator (KDE) on the resulting set.

Based on the many-to-one nature inherent to this problem, we hypothesized that a training set that is more uniformly distributed in 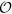 will have better performance in testing due to less redundancy in similar images during training. That is, for a training set that is particularly clustered in 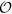, two similar images with disparate pillar sequences may “undo” whatever is learned from the other, resulting in a less effective model overall. Conversely, a training set that is well distributed in 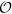 would have more unique images, perhaps mimicking a one-to-one design space, and create better predictions. We tested this hypothesis by assembling 50 randomly generated 150,000 image datasets into a single 7.5 million image super dataset. The corresponding PCA projection of this dataset is constructed and sampled such that each image is isolated within an n-D hypercube of a size defined by a uniform discretization of 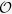 (see Fig. 8(a,b) for a demonstration of this process). This results in a method for selection of training data that is more uniformly distributed in 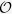 (See Fig. 8(c,d) for examples of such a dataset).

We performed uniform 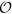-sampling for both RNG and HDMR super datasets, and tested the resulting ≈150,000 image training set with the same data as in the first study, and found no significant change in performance. The median PMR values in testing was 0.79 and 0.81 for RNG and HDMR generated data, respectively, with no significant change in variance. This follows with results from the out-of-sample study, as it was shown that a clearly non-uniform distribution of training data in 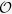 could still make successful predictions.

## Conclusion

There are a large variety of science and engineering problems with a tractable forward problem simulation process and/or a large database which can benefit from a powerful machine learning framework to solve seemingly intractable inverse problems. However, such applications are still at a nascent stage, and important questions related to model choice, training data generation, decision problem formulation, have to be answered to achieve significant impact. In this context, we have shown that intelligent sampling of training data in deep learning models for flow sculpting can greatly improve performance, with the capacity to raise the ceiling on training set size for additional improvement. We also demonstrated deep learning’s capability to effectively make predictions well outside of the trained scope, with additional improvements in out-of-sample predictions through HDMR sampling. Finally, we tested the hypothesis that a training set made artificially uniform in the training data’s image space leads to a more effectively trained model, but found no evidence in support of this claim. In fact, this result meshes well with the out-of-sample testing, where the deep learning model was still capable of successful predictions despite a substantial gap in the image space of the training data. With improved accuracy, high model confidence, and potential for further growth, deep learning will see immediate use for design in flow sculpting, while analogous problems in scientific design can benefit from the lessons of its application here.

## Methods

### Forward Model via Computational Fluid Dynamics

The forward model of simulating a fluid flow shape given a sequence of micropillars follows the experimentally validated method used by Stoecklein *et al*.^16^,^17^, which we briefly outline here. A library of pre-computed deformation maps are created for a set of pillar diameters and locations in the channel, with a constant channel aspect ratio (height/width) and matching flow physics (*Re*). To create a single map, a 3-D velocity field is simulated for a given pillar configuration using an experimentally validated finite element framework. The 3-D domain extends well beyond the pillar (≥6*D*) to allow for the structure-induced deformation to saturate within the domain^3^. Then massless, neutrally buoyant fluid elements are traced through the velocity field to determine the 2-D displacement of fluid in the cross-section of the channel from inlet-to-outlet, defining an advection map for a single fluid deformation. An advection map informs the creation of a square transition matrix, which has a row and column for every discretized cross-section location in the streamtrace. For every starting location of a fluid element in the inlet cross-section, there is a single value of unity in a column indicating 100% likelihood of displacement to that location in the outlet cross-section.

A fluid flow shape at the inlet can be represented as a vector of fluid states in the channel cross-section, ***μ***_**0**_, with zeroes for untracked fluid and ones for the fluid being sculpted. This inlet vector can then be multiplied by the transition matrix to create an outlet vector, ***o***, which represents the deformed fluid flow shape at the outlet. By using binary values for transition probabilities (i.e., 0 or 1), the matrices are extremely sparse, leading to very efficient computation. On modest computing hardware, a single pillar can be simulated in roughly 3 milliseconds^16^. Any number of transition matrices can be multiplied together, each created from an arbitrary pillar configuration, ultimately creating a net transition matrix which produces the deformed shape for the entire sequence. In general, any kind of geometry for flow physics can be implemented within the flow sculpting forward model, e.g., curved channels, steps, non-pillar structures, and multi-fluid flow^40^. However, for the overall prediction to be valid, the flow physics (defined by *Re*) must match for each pre-computed deformation, and the microchannel geometry (defined by the channel aspect ratio) must either also match for each new transition matrix, or additional deformations must be computed to stitch together the step from one channel geometry to another.

### Deep Learning Implementation

The CNN used consists of 2 convolutional layers (40 kernels of size 11 × 11 for the first layer, 100 filters of size 4 × 4 for the second layer), each pooling layer followed by downsampling by 2 × 2 maxpooling, and one fully-connected layer with 500 hidden units (see Fig. 2). The loss function was a negative log-likelihood as defined in Lore *et al*.^18^, and the learning rate was 0.1. Training was performed on sample sizes from 50,000 to 250,000 (with an additional 20,000 validation samples), while testing was performed on 10,000 samples generated through random sampling of 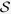. The framework as described above was implemented in the Theano platform^43^, and performed on NVIDIA K20 Tesla GPUs. All experiments were deployed on Iowa State University’s High Performance Computing cluster Cyence, with post-processing and analysis conducted on an NVIDIA Geforce GTX Titan Black.

## Additional Information

**How to cite this article**: Stoecklein, D. *et al*. Deep Learning for Flow Sculpting: Insights into Efficient Learning using Scientific Simulation Data. *Sci. Rep.* **7**, 46368; doi: 10.1038/srep46368 (2017).

**Publisher's note:** Springer Nature remains neutral with regard to jurisdictional claims in published maps and institutional affiliations.
